# Older Adult Movement Assessment Through Rehabilitation Software for Upper Limb Exoskeleton

**DOI:** 10.3390/s26123658

**Published:** 2026-06-08

**Authors:** Angel Camacho, Daniel Celis-Ruiz, Hellen Rivero-Pineda, Mariana Ballesteros, David Cruz-Ortiz

**Affiliations:** 1 Centro de Innovación y Desarrollo Tecnológico en Cómputo (CIDETEC), Instituto Politécnico Nacional, Mexico City 07700, Mexico; acamachol1700@alumno.ipn.mx (A.C.); hriverop1700@alumno.ipn.mx (H.R.-P.); 2 Universidad Politécnica de Quintana Roo, Cancún 77500, Mexico; angelcelis2004048@gmail.com; 3 Medical Robotics and Biosignals Lab, Unidad Profesional Interdisiciplinaria de Biotecnología (UPIBI), Instituto Politécnico Nacional, Mexico City 07340, Mexico

**Keywords:** rehabilitation, older adults, serious games, wrist motion, robotic devices

## Abstract

This work presents a pilot study to analyze the effect of aging on motor performance of young adults (YAs) and older adults (OAs) through wrist movement assessment, using an upper limb rehabilitation robot (ULRR) in passive mode coupled to a maze-solving task serious video game. The proposed approach considers the use of kinematic metrics, such as ROM, path accuracy, and movement smoothness, as quantitative biomarkers that evidence differences between YAs and OAs. An experimental protocol was conducted with 20 participants: 10 OAs and 10 YAs. Standardized wrist movements corresponding to flexion (F), extension (E), radial deviation (R), and ulnar deviation (U) were assessed at each level of the maze. The kinematic analysis was based on metrics for range of motion (ROM), path accuracy, smoothness, and root-mean-square error (RMSE) in trajectory tracking. The results revealed clear differences between the groups: the YAs achieved a greater ROM and made fewer errors on mean (2.167 errors for YAs compared to 6.000 errors for OAs), and showed a lower RMSE, while the OAs showed greater smoothness in their movements, because the YAs exhibit greater variability and disturbances in movement when correcting and controlling their movements to achieve good performance, reflecting more precise motor control and a greater capacity for error correction during movements with trajectory constraints.

## 1. Introduction

Nowadays, the rising number of older adults (OAs) poses a significant challenge for health systems, mainly in the geriatric field [1]. By 2050, elderly individuals will make up approximately 16% of the world’s population, doubling the current level [2]. This is especially relevant for healthcare systems. As the elderly population grows, the demand for comprehensive medical care rises, creating a crucial challenge [3]. In this context, the World Health Organization (WHO) projects that from 2015 to 2050, the proportion of OAs will rise from 12% to 22%. Then, by 2025, 80% of this population will be living in low-income countries [4], resulting in a major challenge for healthcare systems but also for the medical technological industry, since the need for specialized equipment and treatments tailored to geriatric care will be required [1,3].

In this context, as humans age, they experience intrinsic morphological and metabolic changes resulting in neuromuscular and sensory diminution capability, usually produced as the consequence of decreased nerve conduction velocity, loss of muscle mass and strength, aside from alterations in sensory integration, among others [5], directly affecting their motor skills [6]. As a consequence, aging is associated with diminished bilateral motor control, characterized by reduced coordination between limbs and increased variability in bilateral movements, which can compromise the independence of the OAs [6,7].

Notice that mobility is a key indicator of aging and health status in OAs, since performing any motor activity requires cognitive and neuromuscular skills to enable visuomotor coordination necessary for planning and executing movement [8]. Then, the rehabilitation field has been studying and offering potential solutions to address sensory and motor limitations and promote independence in activities of daily living (ADLs) among OAs [9]. However, conventional rehabilitation tests have certain limitations: they do not always rely on objective quantitative metrics and instead rely largely on the specialist’s qualitative assessment [10]. As a result, there has been growing interest in developing methods to assess motor performance quantitatively, promoting the implementation of personalized strategies as future treatments.

Currently, there has been a surge in methods to assess motor performance and advance motor rehabilitation strategies for OAs, focusing mainly on a better understanding of the effects of aging on motor function [4]. Several studies have centered on analyzing the differences between young adults (YAs) and OAs as a preliminary approach, since OAs experience declines in motor control efficiency and in the ability to perform precise, coordinated movements during functional tasks compared with YAs. Therefore, analyzing these differences could provide insight into the evolution of motor function variability and the individual’s ability to perform ADLs during aging [11,12].

Several studies have focused on comparing motor sequence performance and learning in YAs and OAs [13,14], particularly in the upper limb, the most affected extremity. In [15], the authors analyzed motor task performance in YAs and OAs during the acquisition of movement sequences. The study revealed differences between both groups in movement duration, degrees of freedom (DoF) range, sensory feedback integration, and control processes. Nevertheless, after identifying relevant factors that influence motor performance, the variability in motor response execution among OAs remain incompletely understood, since the process involves dynamic interactions between cognitive and physiological processes. Then, complementary studies are required for a precise understanding.

As consequence, standardized clinical scales used in rehabilitation has been implemented to assess upper limb movement (ULM) in YAs and OAs [16,17,18,19], such as the Fugl–Meyer Assessment (FMA) and the Arm Research and Action Test (ARAT) [20,21]. Nevertheless, these approaches remain primarily qualitative and do not fully capture the complexity of motor execution, needing the inclusion of quantitative indicators to improve the accuracy and reproducibility of traditional indices and to limit errors arising from direct observation [22]. While these tools can be supplemented with motion analysis technologies to quantify motor performance, standardized metrics for these technologies remain lacking. To address this, some studies have used multimodal instrumented systems that enable the objective assessment of kinematic, dynamic, and neuromuscular variables in YAs. Specifically, kinematic analysis has focused on the use of inertial measurement units (IMUs) [23], motion capture systems [24], and robotic rehabilitation systems (RRS) [25].

In this context, despite the existing concept of passive RRSs, which do not necessarily have actuators but instead use mechanisms such as springs, dampers, or guide mechanisms to restrict movement or guide paths. Also, the concept of active RRSs (or only RRS) has been introduced for assessing ULM. These systems are conventionally used to produce motion or exert force via actuators (electric or pneumatic, among others), which are coupled to electronic instrumentation that measures variables of interest. Nevertheless, approaches focused on assessing upper limb have promoted its implementation perating in passive mode [26,27], especially when the study and the proposed tasks are centered on the evaluation of kinematic parameters.

For instance, the study reported in [26], used different experimental wrist devices to determine kinematic parameters for assessing ULM. In this particular case, even though the system includes brushed direct current motors in its electronic instrumentation, for the proposed task, the system operated in a passive mode, that is, an unpowered backdrive mode when assessing movement, relying on intrinsic device transparency. Note that in the works [26,27,28], no complex tasks are required to obtain relevant information in assessing ULM, when the considered RRS meets or exceeds the range of motion (ROM) requirements for ADLs, which is the first requirement for assessment. Then, most studies in the literature consider basic wrist movements such as flexion/extension (WFE) and radial deviation/ulnar deviation (WRU) as the first approach to obtain relevant data for assessing ULM.

To sum up, the use of RRS for assessing ULMs in adults has motivated the use of assistive exoskeletons to guide and quantify motor performance using kinematic, dynamic, and movement-planning metrics during training [11]. However, none of the mentioned approaches have been used to study and analyze the effect of aging on ULM in OAs, making this an open problem in the rehabilitation field.

On the other hand, despite advances in RRSs, their effectiveness largely depends on the quality of interaction between the user and the device, which has driven the development of Graphical User Interfaces (GUIs) and feedback systems to improve communication between the user and robotic system, promoting greater awareness and control over the cognitive and motor processes involved in rehabilitation [24,29]. Alongside software and GUIs, the concept of serious video games, defined as those whose objectives go beyond entertainment, has surged in the rehabilitation field, and particularly in assessing ULM [30]. Notice that the purpose of serious video games in rehabilitation, also known as rehabilitation games, is to stimulate the user’s visuomotor coordination and to involve a willingness to actively engage in exercise [31,32].

In that sense, rehabilitation games combined with some protocols for assessing movement have been implemented as a first approach to evaluate the ULM [33,34,35], such is the case of the work [33], where a framework focused on rehabilitation and assessment of the upper limb motor function based on serious games is presented. However, although a range of protocols for assessing ULM can be found in the literature, their potential combined with the rehabilitation games has not been explored in detail, as is the case with protocols as tests of object-directed movements [36], throwing tasks [37], finger tapping [38], and maze-solving task.

In particular, regarding the last of the mentioned protocols maze-solving task, and according to Cienfuegos et al. [39], this task was implemented in which the user performed movements within a controlled, structured environment. However, this study could be complemented by the use of serious games to motivate patients to perform their rehabilitation routine. This type of task allowed for the assessment of fine motor performance (characterized by hand and wrist movements), which is often one of the first functions to be affected and is key in the initial stages of analysis or rehabilitation in OAs [40]. Additionally, it facilitates the objective quantification of movement, as the maze-solving task provides a defined path that requires continuous integration of visual information with movement planning and motor control within the constraints of the task.

Based on the previous arguments, this work presents a pilot study to analyze the effect of aging on motor performance of YAs and OAs through wrist movement assessment, using an upper limb rehabilitation robot (ULRR) in passive mode coupled to a maze-solving task serious video game. The proposed approach considers the use of kinematic metrics, such as ROM, path accuracy, and movement smoothness, as quantitative biomarkers that evidence differences between YAs and OAs.

To this end, throughout the study, a set of experiments was conducted with participants with no musculoskeletal conditions affecting the upper limb who performed wrist ROM assessments and wrist-pointing tasks through maze-solving using a wrist-and-forearm exoskeleton, the ArMexo (Instituto Politécnico Nacional, Mexico city, Mexico) [41]. YAs and OAs perform these tasks to evaluate the differences between the two populations. The decision to include YA and OA participants without upper-limb musculoskeletal conditions limits the presence of additional motor deficits (e.g., poor coordination, often present in several musculoskeletal conditions) in the metrics obtained, thereby reducing the risk of confounding the movement smoothness assessment and clearly evidencing differences between the two populations.

As a starting point for evaluating the effect of aging on wrist movement assessment, the experiment addresses three aims: first, to quantify the diminution of wrist joint ROM; second, to quantify the user’s ability to maintain fine motor control within the limits imposed by the video game by comparing the user’s cursor trajectory with the ideal trajectory; third, to quantify the movement smoothness measured via spectral arc length (SPARC). The outcomes of this experiment are used to inform guidelines for evaluating, quantitatively, the effect of aging on motor performance in YAs and OAs.

The main contribution of this study lies in exploring the potential of combining RRSs with maze-solving tasks to evaluate, in a quantitative manner, differences between YAs and OAs, an approach not previously reported in the literature.

## 2. System Description

**Figure 1:**
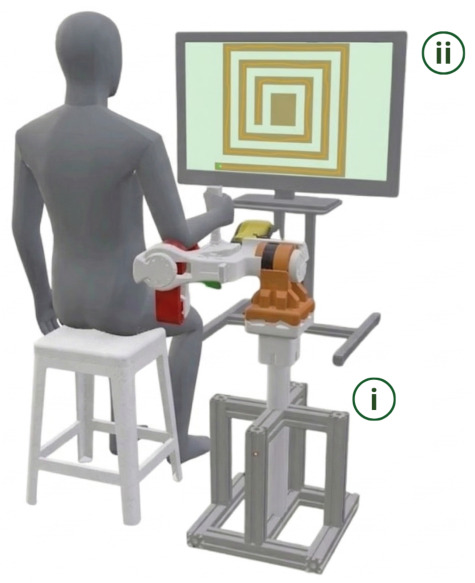
Diagram of the system integration: (**i**) hardware; (**ii**) software.

The proposed system consists of two subsystems: the hardware, whose main component is an ULRR (see Figure 1i) and a software consisting of a serious video game with interactive mazes (see Figure 1ii). The interaction between the two subsystems enables the online collection of kinematic and kinetic signals (such as joint range, torque, and movement speed) from the wrist while the user performs a movement protocol via the interactive game.

### 2.1. Hardware

**Figure 2:**
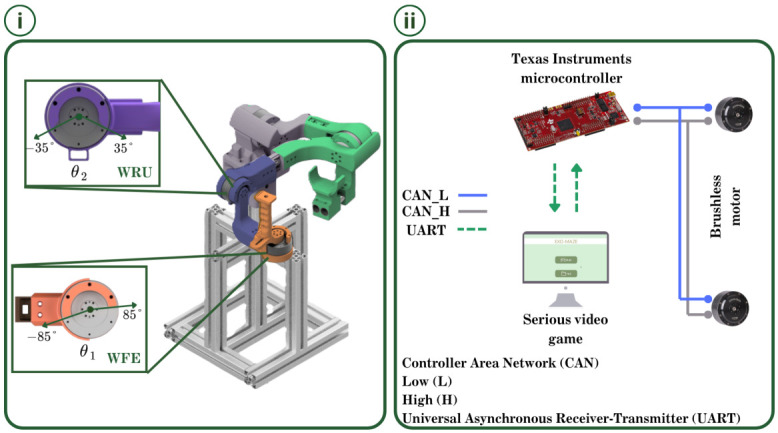
Hardware diagram. (**i**) ULRR: mechanical structural design. (**ii**) Electronic instrumentation.

This subsystem consists of two elements: the ArMexo, which is a ULRR, and its corresponding electronic instrumentation, as shown in Figure 2.

For this study, the ULRR considers four DoF: elbow flexion/extension, forearm supination/pronation, WFE, and WRU. However, due to the design of the serious video game, only WFE and WRU are considered. The length of each section of the ULRR (arm length, forearm length, hand length, and hand width) was determined based on the anthropometric dimensions of the Latin American population [42]. A carbon fiber-reinforced polylactic acid filament was used to manufacture the structure. The mechanical design includes the ROM for each joint based on biomechanical data of the upper limb, and safety measures, such as mechanical stops, have been included to mitigate risks to users (See Figure 2i).

Regarding the ArMexo systems, their motors can measure angular position, angular velocity, and torque. All the data are stored in 16-bit frames in Big Endian format. These are collected at a sampling rate of 100 Hz, allowing human kinematic movement to be recorded without aliasing [43]. On the other hand, the second element of the hardware, which is the electronic instrumentation of the ULRR, is mainly integrated by a Texas Instruments TM Launchpad F28379xD microcontroller that implements the CAN Bus communication protocol to read the motor signals and send commands to each motor, as shown in Figure 2ii. Further design details can be found in [41].

Notice that despite the complex structures of ULRRs such as the ArMexo, several studies in the literature consider partial use of their structures or functions, depending on the task and the parameters to be analyzed in the proposed approach, especially when the system operates in passive mode that is, unpowered backdrive mode when assessing movement, relying on intrinsic device transparency [26,27]. Indeed, according to the literature, passive mode has been several used when the objective is to evaluate kinematic parameters, such as the case of this study.

On the other hand, the choice of considering only WFE and WRU movements in this work was based on the movement assessment perfomed in previous studies, where single movements are evaluated to have less compensation artifacts, basic biomarkers can be determined, and it represents a lower cognitive and physical demand to the users [26], in this case including OAs. Several studies have analyzed measures of ULM smoothness and coordination. Nevertheless, these analyses have not been fully reported for the wrist. Moreover, wrist-pointing movements have been observed to be less smooth and more variable than elbow and shoulder pointing movements, making them more interesting to analyze in recent studies.

In addition, although most studies of human voluntary movement control have focused on whole-arm reaching movements, wrist rotation is critical for normal upper limb function. By orienting the hand, the wrist connects reaching movements to grasping movements, allowing the hand to engage with objects and manipulate them. According to [44], there was a need to characterize and understand the fundamentals of unimpaired wrist behavior. However, as the OAs population increases, there is a need to characterize differences between YAs and OAs.

In that sense, notice that even when it seems that the proposed task contrasts with the sophistication of the system, the considered test includes tasks requiring WFE, WRU, and combinations, which, as can be corroborated in the literature [26,44], is a starting point considered in various studies.

Building on previous ideas, the proposed video game consists of a maze-solving tasks with movement restrictions, designed to assess motor planning in response to the various cognitive and motor challenges at each level. Then, each proposed maze task at different levels requires WFE, WRU, and combinations of the two. Here, it is important to emphasize that the results offer a different approach from previous studies, which are mainly focused on wrist assessment in YAs.

### 2.2. Software

For this study, this element, which comprises high- and low-level layers, was designed as a serious video game in which the user performs different maze-solving tasks through a ULRR. Notice that for this study, this element represents a controller experimental task rather than a functional simulation, since the proposed approach allows us to obtain biomarkers that enable us to understand, in a quantitative manner, the effect of the aging process on wrist assessment movements; moreover, the obtained results could serve as a basis for their future application in improving conventional rehabilitation routines.

Then, as previously mentioned, this element is divided into two main layers: a high-level GUI for user interaction, data acquisition, visualization, storage, and the serious video game, and a low-level Firmware embedded on the development board (microcontroller) for online acquisition and actuator managemen in the ArMexo system.

#### 2.2.1. High Level Layer

**Figure 3:**
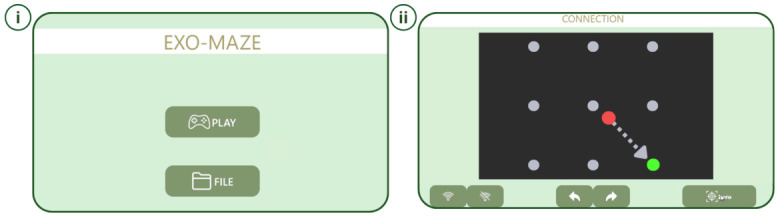

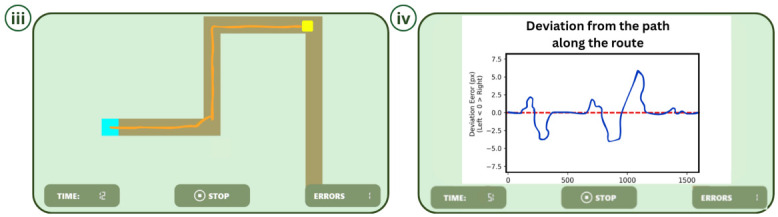
Main sections of the software designed (**i**) Main menu, (**ii**) Calibration window, (**iii**) Game window, and (**iv**) Results window (The graph shown is for illustrative purposes only).

The high-level layer integrates the serious video game and its interface. The designed GUI has four main sections: (1) Main menu (see Figure 3i), (2) Calibration window (see Figure 3ii), (3) Game window (see Figure 3iii) and (4) Results window (see Figure 3iv). The following section provides a detailed description of the software development process and how it is integrated with the ULRR.

#### 2.2.2. Firmware

The low-level layer consists of a program embedded in a TMS320F28379D LaunchPad™ development kit (Texas Instruments, Dallas, TX, USA), as mentioned earlier. To ensure the accuracy of the collected data, the firmware focused on two steps, as shown in Figure 1ii:

**Data acquisition:** This step is in charge of reading estimated data from the motor encoder via the CAN Bus communication protocol.

**Data packaging:** This second step is for the packaging of bits collected from each motor’s encoder for subsequent transmission to a desktop computer via a UART serial bridge.

## 3. Serious Game Description

The QtDesigner 5 tool was used to design the main sections of the GUI. In this stage, Python (v3.12.4) was used to develop the GUI for signal acquisition, processing, visualization, and storage. Both tools offered great flexibility in system design thanks to their extensive libraries and their capabilities for developing complex interfaces [45].

As mentioned before, the serious video game consists of four main sections, described below:

**Main menu:** This section corresponds to the start of the game. The window has only two buttons to provide a user-friendly design. The **Start button** allows execution of the next sections, and the **File button** permits the user to define the directory where the collected data will be stored (see Figure 3i).

**Figure 4:**
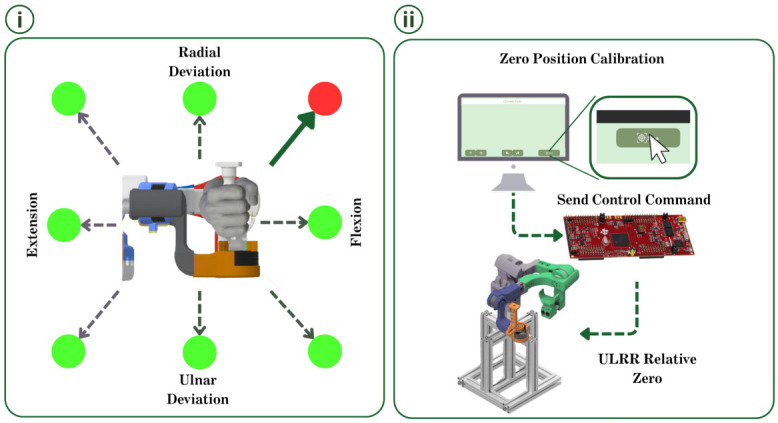
Calibration window diagram: (**i**) points to reach during calibration; (**ii**) pre-calibration command sequence.

**Calibration window:** In this section, the user performs a series of movements using the WRU and WFE movements mentioned above to reach the points displayed on the screen (see Figure 4i). This process recorded the ROM performed by the user. During the game, only 75% of the maximum reached in the calibration session will be used to prevent fatigue and difficult movements during the session.

Before the calibration stage, a control command is sent to the microcontroller in order to reset the encoder registers to a relative zero value (see Figure 4ii), ensuring that the physical position of the ULRR aligns with the participant’s joint zero point.

**Game window:** This section displays the EXO-MAZE game, which shows interactive mazes designed according to the proposed protocol (described below). The user must navigate it using the WRU and WFE movements until all the mazes are completed.

**Figure 5:**
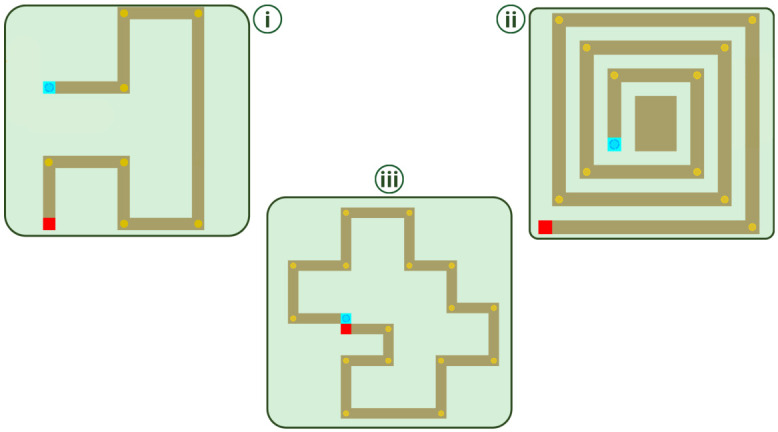
The three-level path: (**i**) Level 1, (**ii**) Level 2, (**iii**) Level 3. Blue square: beginning of the maze, Yellow coins: movement change, Red square: finish of the maze.

The video game has three consecutive levels (see Figure 5) that differ in the number of directional changes the user must perform. For this study, each level considers different dimensions in pixels for the mazes: maze level 1 (990 × 990), maze level 2 (765 × 810), and maze level 3 (1755 × 1440). Nevertheless, each maximum in pixels corresponds to 75% of each user’s ROM in WFE and WRU, respectively.

The first level (see Figure 5i) features two turns in each direction (up, down, left, and right), each corresponding to a corner of the path where a coin is located that the user must touch; thus, by the end of the level, the user must have passed through all seven coins on the path. In the second level (see Figure 5ii), the number of coins increases to eleven, so that the user must change direction three times to complete the level. For the third and final level (see Figure 5iii), sixteen coins were placed, corresponding to four changes in direction on each side along the course. It is important to note that this video game is a controlled, experimental task designed for the standardized quantification of kinematic indicators for the assessment of motor performance; these values will be applied in future studies as standardized values for implementing rehabilitation routines.

**Figure 6:**
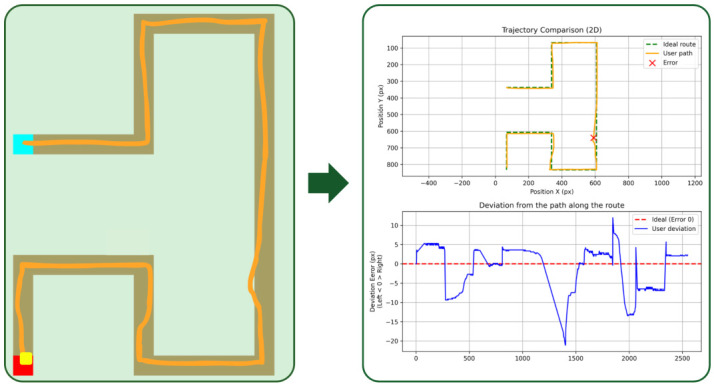
Graphical representation of collected data. Blue square: beginning of the maze, Yellow square: cursor of the user, Brown line: maze path, Orange line: user path, Red square: finish of the maze.

**Results window:** This section is enabled once any level is completed. It consists of a pop-up window that displays a comparative graph of the ideal route with the route taken by the user, as well as markers where the user deviated from the path (see Figure 6). The ideal path consists of connecting the midpoints of each linear segment, serving as the movement reference that the user must replicate.

Once the user has completed all the levels, the collected data will be saved to the previously specified directory.

## 4. Experimental Setup

This study conducted a series of experiments to assess the effect of aging on movement smoothness during goal-directed wrist-reaching movements within a limited ROM, using a maze task. In particular, for the test, YAs and OAs without musculoskeletal conditions affecting their upper limbs performed point-to-point reaching movements using the ArMexo (in passive mode) to move a cursor on a screen across different maze scenarios. Participants used both WFE and WRU deviation to complete each maze level test.

For the study, a protocol and experimental setting were established to improve the study’s reproducibility and minimize artifacts as much as possible. As the first stage of the study, anthropometric measures from the users were collected from both arms. The considered anatomical segments include arm length, forearm length, forearm circumference, arm circumference, and palm length and width. In addition, general information about the participants, such as age, gender, occupation, and frequency of physical activity, was collected.

The test was conducted in a 3.5 m × 3.5 m room, with a 55-inch screen positioned 1 m above the floor and 1.5 m from the user. Lighting was kept constant, and black curtains were installed to prevent external distractions. All tests were performed with the participants on a wooden floor to create an environment isolated from electromagnetic interference and to prevent electrostatic discharges. During the task, participants remained seated in a chair with lumbar support, maintaining an upright posture with their feet firmly planted on the floor and their torso stabilized to prevent compensatory movements, as shown in Figure 1.

### 4.1. Participants

As part of the technical validation and pilot study of the proposed approach, twenty participants of Mexican nationality, divided into two groups: the first group consisted of 10 OAs (six women and four man) with an age of 64 ± 6 years, and the second group of 10 YAs (seven men and three woman) with an age of 24 ± 5 years, all of them right-handed. The sample size (N = 20) was determined in accordance with methodological recommendations for pilot proof-of-concept studies in rehabilitation engineering, where the primary objective is to assess the system’s stability and the sensitivity of the metrics prior to larger-scale clinical trials [46]. Participants were selected based on inclusion and exclusion criteria stipulating that OAs had to be at least 60 years old, YAs between 18 and 40 years old, and none of them could have reported any musculoskeletal conditions affecting the upper limbs. All individuals provided written informed consent before their participation, affirming their voluntary involvement and understanding of the study procedures.

The protocol with number SIP-20260076 was approved by the Secretaria de Investigación y Posgrado del Instituto Politécnico Nacional (IPN), ensuring it met the ethical standards of the Declaration of Helsinki. Experimental sessions were subsequently conducted at the Medical Robotics and Biosignals Laboratory of the National Polytechnic Institute in Mexico City, in accordance with the approved protocol.

### 4.2. Task Description

Subjects were seated with the right forearm resting in the para-sagittal plane (∼25 cm from the midline) on the support of the ArMexo system (the shoulder was abducted ∼40° and flexed ∼35°, and the elbow was flexed ∼60°). Participants were then asked to manipulate the ArMexo handle to move the cursor on the screen. The proposed task comprised two sections: the calibration stage (ROM assessment) and the test (wrist pointing). In the first stage, their ROM was assessed, whereas in the second, their movement smoothness (measured with SPARC) was evaluated as they performed wrist-pointing movements across different maze scenarios.

#### ROM Assessment

Before taking a test, the user underwent a calibration procedure to adjust the game to the appropriate ROM in order to determine each user’s workspace and set it as their limit in the game.

**Figure 7:**
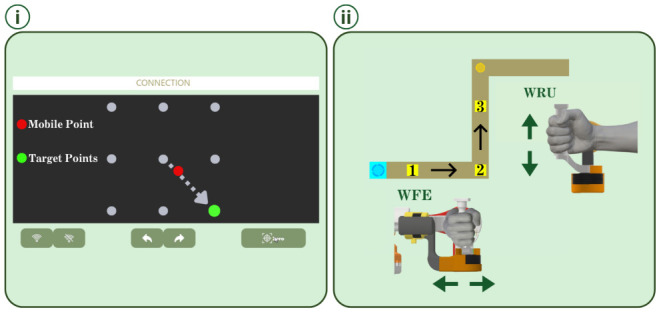
Game protocol and software calibration routine. (**i**) ROM assessment task; (**ii**) Wrist pointing task.

During the calibration routine, as shown in Figure 7i, standardized movements based on the FMA framework were performed, consisting of reaching eight points distributed every 45° in a clockwise direction. Although the FMA is traditionally applied to neurological populations, its use in this study with healthy subjects serves as a measure of methodological standardization, with the aim of establishing appropriate values for each participant [20].

Following the methodology established previously, the calibration procedure required the user to reach eight points spaced every 45 degrees apart in a clockwise direction; the user had 5 s to attempt to reach each point and return to the center, allowing for the collection of their ${\mathrm{ROM}}_{max}$. This routine was repeated twice (see Figure 7i).

Since the protocol proposed in the video game results from combining these movements, a maximum range was established for the ROMs recorded for each participant. To ensure a comfortable and safe interaction for the participant, a standardized ROM (${\mathrm{ROM}}_{std}$) was proposed, consisting of 75% of the mean of each participant’s ${\mathrm{ROM}}_{max}$. This was done with the intention of preventing mechanical fatigue and pain at the joint limits.

### 4.3. Wrist Pointing

The video game was divided into three levels, each designed so that the user would make the same number of movements in each direction (up, down, right, and left), in order to ensure equal performance in accordance with the movements permitted by the WRU and the WFE (see Figure 7ii).

**Figure 8:**
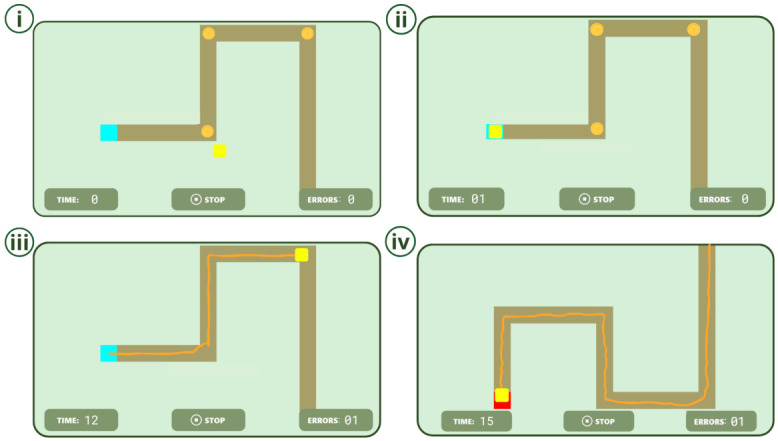
Game protocol. (**i**) Start of the game, (**ii**) 2-Second wait, (**iii**) Game in progress, and (**iv**) End of the game. Blue square: beginning of the maze, Yellow square: cursor of the user, Brown line: maze path, Orange line: user path, Red square: finish of the maze.

Once the game starts, the user is automatically positioned in the center of the screen in all three levels (see Figure 8i). The user must then move to the starting point (blue square) of each level and remain there for 2 s to begin the game (see Figure 8ii). After doing this, the game initializes all its parameters (time, errors, coins collected, and signals collected). To begin the path, the user must follow the marked path, taking care not to reach the boundaries, while collecting all the coins found at the corners of the path (see Figure 8iii). The design of the maze-type task was based on previous studies that have shown that maze navigation games allow for the indirect estimation of motor control indicators such as anticipatory planning, movement prediction, and real-time error correction and have been used as analogs for complex ADLs such as driving or navigation, especially in OAs [40,47]. Finally, to complete the level, the user must reach the end of the path, marked by a red square (see Figure 8iv).

### 4.4. Data Verification

In order to verify that the data collected in each test were consistent and valid for post-processing, they were subjected to an integration test at the end of each session. The validation criteria established in this document were: a stable sampling frequency of 90–100 Hz, all coins having been captured, and a maximum data loss rate of 3% of the total data.

To calculate the sampling frequency of the collected data, a sampling scheme based on the time difference between successive samples (Δt) was implemented using the Equation (1), with the intention of ensuring a constant sampling period in the data. Here, the sample rate is denoted as follows

(1) $$f_{s}=\frac{1}{\Delta t},$$

where

$$\Delta t=t_{n}-t_{n-1},$$

with

- $f_{s}$: Sample rate
- $t_{n}$: Timestamp of the current sample
- $t_{n-1}$: Timestamp of the previous sample
- Δt: Time interval (sampling period)

## 5. Data Management

This section explains the organization and handling of raw data, detailing its storage, segmentation, and processing. At this stage, data integrity is ensured to allow for a standardized comparison between the two study groups.

### 5.1. Database

**Figure 9:**
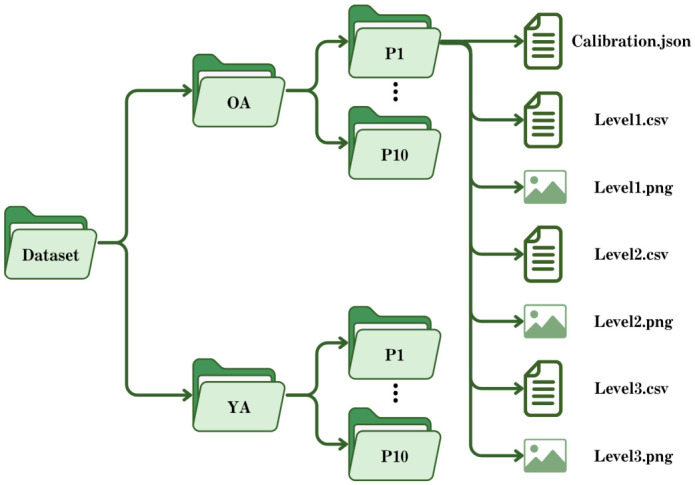
Database structure.

The data was stored in a hierarchical structure, as shown in Figure 9, to organize both groups for processing. The structure is primarily divided into OAs and YAs; within each division, there is a folder for each participant. Within each folder is a JavaScript Object Notation (JSON) file containing the metrics collected during the calibration stage (ROM for cardinal and intercardinal directions), a Comma-Separated Values (CSV) file containing the kinetic and kinematic data for each level, and an image of the comparative graph resulting from the results section for each level.

The following data was stored in the CSV file generated for each level:

- **Time_s**: Corresponds to the absolute timestamp from the system.
- **WFE data**: Kinematic (velocity in degrees per second and position in degrees) and kinetic (torque in Newton-meters) signals corresponding to the WFE movement.
- **WRU data**: It also stores kinematic (velocity and position) and kinetic (torque) signals corresponding to the WRU movement.
- **Number of errors**: Corresponds to the number of times that the user deviated from the path.
- **Coins captured**: It is a counter for coins collected along the way.

All of these metrics and indicators collected throughout the session were used for data processing, as described below.

### 5.2. Segmentation

As mentioned before, in order to facilitate the segmentation of each movement performed at the different levels, markers were placed at all corners of the game to indicate the exact moment when a change in direction occurs. Using these time markers, it was possible to divide each movement into three levels, and the resulting segments were subsequently analyzed. For this study, the total set of performed movements in each level is given by Equation (2), that is,

(2) $$M=\overset{n}{\underset{i=1}{⋃}}\;\left[\;t_{i},\;t_{i+1}\;\right],$$

where

- *M*: Total set of movements performed on the level
- $t_{i}$: Timestamp associated with the coin in the corner *i*
- *n*: Total number of coins on each level

## 6. Motion Analysis

This section describes the kinematic metrics used to assess motor control and performance in participants from both experimental groups. For this study, the metrics of ROM, Root Mean Squared Error (RMSE), and SPARC were selected because they quantify spatial interaction ability, precision in trajectory execution, and neuromotor efficiency, respectively. Notice that these parameters are relevant to evaluate the differences in wrist assessment movements of YAs and OAs, since better values in those parameters imply greater autonomy and safety when performing ADLs.

In addition, according to the literature [7,48,49], the selected metrics may be directly related to physiological meaning and to conventional metrics used in the clinic, as shown in Table 1.

### 6.1. ROM

ROM is a key parameter for quantifying joint mobility, as it defines the ${\mathrm{ROM}}_{max}$ a joint, in this case, the wrist, can achieve. This metric is reflected in ADLs such as reaching, carrying, lifting, pushing, pulling, and manipulating objects—activities related to stiffness and mechanical and functional limitations [50].

As mentioned in the Section 4, the ${\mathrm{ROM}}_{std}$ values for each participant were determined during the calibration phase in this study. During this stage, Motor 1 was linked to the WFE movement, while Motor 2 was associated with the WRU deviation. Finally, the data obtained was stored in each subject’s individual JSON file.

### 6.2. Path Accuracy

Trajectory accuracy is a metric that assesses the user’s ability to maintain fine motor control within the limits imposed by the video game. This metric relates to how aging affects motor stability, due to the inability to maintain stable movement, which indicates a decline in the neurophysiological system [51].

For this indicator, the user’s cursor trajectory was compared with the ideal trajectory defined in the Section 3. The RMSE was used to evaluate the mean deviation between the trajectory performed by the subject and the reference trajectory using Equation (3). An RMSE value close to zero indicates high precision of the actual trajectory relative to the ideal trajectory, while a high RMSE suggests difficulties in fine-tuning the trajectory. For this study, the RMSE is defined as follows

(3) $$RMSE=\sqrt{\frac{1}{n}\sum_{i=1}^{n}\left[{\left(x_{i}-x_{ref}\right)}^{2}+{\left(y_{i}-y_{ref}\right)}^{2}\right]},$$

where

- $x_{i},y_{i}$: Coordinates (x, y) obtained from the user’s path.
- $x_{ref},y_{ref}$: Coordinates (x, y) obtained from the reference path.
- *n*: Total number of samples.

In addition, the number of collisions was recorded as the number of times the cursor coordinates exceeded the corridor width (45 pixels). For this purpose, the different elements of the map, such as corridors, walls, coins, and the goal, were created as geometric shapes, each identified by its own name. Collisions were detected using a Python function that identifies intersections between the cursor and geometric figures. This metric complements the RMSE, as it allows us to determine whether the trajectory errors were minor, consistent deviations, or sharp deviations that took the user outside the permitted work area.

### 6.3. Movement Smoothness

The smoothness of motion was computed using the SPARC metric, which is defined in the frequency domain. This indicator assesses overall fluidity and coordination of movement and is commonly associated with FMA for the upper limb [48]. This metric considers the frequency-normalized Fourier magnitude spectrum of the velocity profile, where the SPARC metric is defined as follows.

(4) $$\begin{array}{c} \mathrm{SPARC}≜-\int_{0}^{\omega_{c}}\sqrt{{\left(\frac{1}{\omega_{c}}\right)}^{2}+{\left(\frac{d\hat{V}\left(\omega \right)}{d\omega }\right)}^{2}}\;d\omega ;\;\hat{V}\left(\omega \right)=\frac{V(\omega )}{V(0)},\\ \omega_{c}≜\min\left\{\omega_{c}^{\max},\;\min\left\{\omega \;|\;\hat{V}\left(\omega \right)<r\right\}\right\}, \end{array}$$

where V(ω) represents the Fourier magnitude spectrum, and $\hat{V}\left(\omega \right)$ denotes the normalized spectrum with respect to its zero-frequency component V(0). The interval $\left(0,\omega_{c}\right)$ defines the frequency band of interest, where $\omega_{c}$ is the cut-off frequency, bounded above by a predefined limit $\omega_{c}^{\max}$. As recommendation by [52], in this study $\omega_{c}^{\max}$ was set to 20Hz and a normalization threshold of r=0.05 was adopted. To analyze the segmented data, signals were classified based on movements generated at each maze level. Signal processing included a fourth-order Butterworth low-pass filter with a cutoff of 30 Hz to reduce high-frequency noise. The SPARC was computed based on the group of movements at each maze level, with signals analyzed individually for each participant. Subsequently, we calculated the mean and standard deviation (SD) movement for each group, and the signals were interpolated and normalized to a standard time scale, enabling comparison between YAs and OAs.

## 7. Results and Discussion

In this section, we analyzed the 720 movements recorded by the developed system, corresponding to the performance of the three levels by 20 participants (10 YAs and 10 OAs). The movements evaluated include F, E, R, and U of the right wrist. These movements were treated as within-subject repetitions to reduce the influence of natural variability in trajectories and obtain more representative and reliable kinematic measures of each participant’s motor behavior. The results of the metrics obtained and a comparison of the performance between the two groups are presented below.

### 7.1. ROM Evaluation

The values obtained in the ROM assessment, presented in Table 2, show that YAs tend to maintain a greater ROM in wrist movements, while OAs exhibit a slight diminution compared with the mean anatomical ROM, particularly in R movement; however, in the F, E, and U movements, ROM was similar between the two groups. This difference, which in the cases of R and E movements exceeds 2 and 3 degrees, respectively. This is consistent with previous findings indicating that the wrist ROM is among the first to be affected by age or inactivity [53].

### 7.2. Path Accuracy Analysis

The accuracy of trajectory tracking was evaluated by calculating the RMSE from a comparison between the reference path and the user’s path. The mean values for each level and group are presented in Table 3. It should be noted that, to ensure all participants could complete the levels, the workspace was mapped to 75% of each subject’s ${\mathrm{ROM}}_{max}$ and normalized to pixels to allow for comparison of the RMSE.

**Figure 10:**
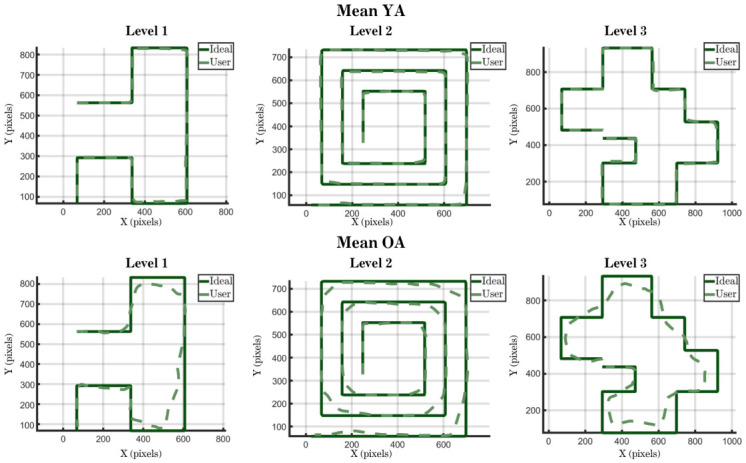
Comparison of the mean path taken by each group with the ideal path.

Analysis of the results shows that the mean RMSE in the OA group is approximately three times higher than in the YA group. Figure 10 illustrates the deviation of the mean trajectory followed by the OAs group from the ideal trajectory. This can be attributed to various neurophysiological factors. On the one hand, it has been reported that changes in motor coordination during aging are linked to a disorganization of the central nervous system [54]. On the other hand, this increase in error is associated with less precise feedforward motor control in the OA population. This deficiency in initial motor programming generates trajectories that deviate significantly from the ideal path, resulting in the high RMSE values observed [55].

Table 4 presents the mean number of collisions recorded for both groups at each level; it shows that the OAs had approximately three times as many collisions as the YAs. In light of the previous analysis, this performance can be linked to deficiencies in initial motor planning. This limitation causes the user to make abrupt movements in an attempt to compensate for initial deviations, resulting in trajectories that deviate significantly from the ideal path [55].

Furthermore, various studies report that aging leads to impairments in the proprioceptive system. As a result, passive motion detection thresholds in the OAs are significantly higher than in the YAs. This decrease in sensory acuity prevents them from perceiving small deviations in trajectory before a collision occurs [56].

### 7.3. Movement Smoothness Analysis

Table 5 presents the smoothness values for maze Level 1, obtained from the analysis of eight movements using the SPARC metric. In the OA group, the lowest SPARC values were observed for all movements compared to the YAs, with a minimum difference of 0.004 and a maximum of 0.021.

For Level 2 analysis, after analyzing twelve movements, the OAs group obtained higher SPARC scores for the R and F movements; the difference between each movement ranged from a minimum of 0.012 to a maximum of 0.070, as shown in Table 6.

Finally, Table 7 presents the results for Level 3. A total of 17 movements were analyzed, in which the OAs showed higher SPARC values in all movements except F, with a minimum difference of 0.007 and a maximum of 0.022.

**Figure 11:**
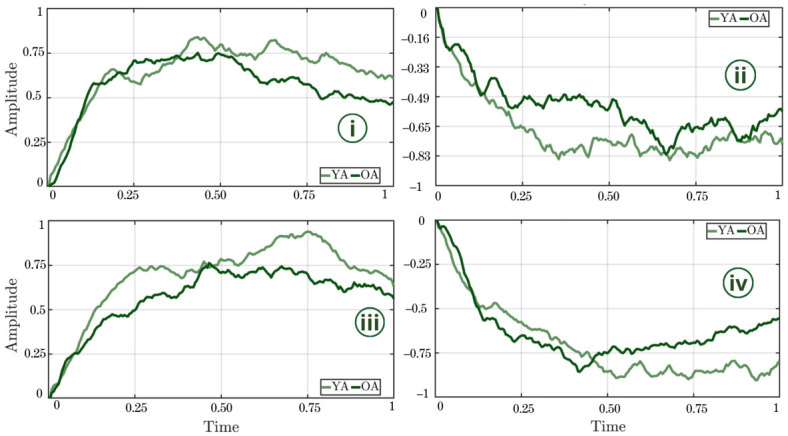
Normalized mean velocity profiles for wrist pointing movements through each DoF in the OA and YA groups at Level 1: (**i**) R, (**ii**) U, (**iii**) E, and (**iv**) F.

**Figure 12:**
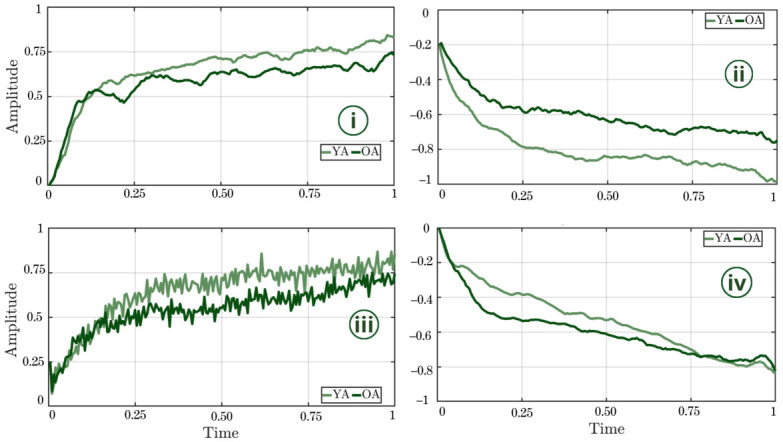
Normalized mean velocity profiles for wrist pointing movements through each DoF in the OA and YA groups at Level 2: (**i**) R, (**ii**) U, (**iii**) E, and (**iv**) F.

**Figure 13:**
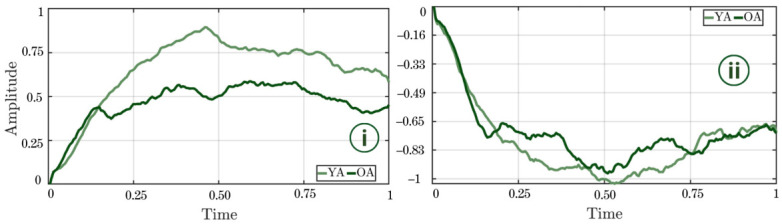

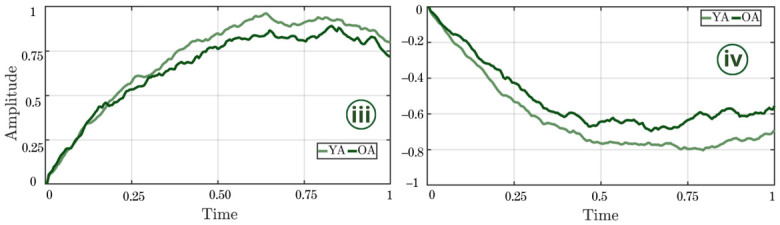
Normalized mean velocity profiles for wrist pointing movements through each DoF in the OAs and YAs at Level 3: (**i**) R, (**ii**) U, (**iii**) E, and (**iv**) F.

Figure 11, Figure 12 and Figure 13 show the velocity profile curves obtained after processing and segmenting the signals corresponding to each movement performed by each subject. The velocity profiles allow us to identify the smoothness of the participants’ motor performance. Smooth movement is represented by a bell-shaped curve, with gradual acceleration to a maximum velocity, followed by gradual deceleration as the movement approaches the target [52]; however, complex tasks involving trajectory adjustments and corrective motor actions can result in velocity profiles with intermittent fluctuations [57,58]. In this case, due to the nature of the video game, the velocity profiles are expected to exhibit intermittent behavior without deceleration, since navigating the maze involves chained movements, continuous trajectory adjustments, and motor microcorrections that require participants to exercise more precise motor control in order to complete the task. Currently, there are time-domain and frequency-domain metrics for evaluating smoothness. SPARC is a metric that evaluates the frequency domain, enabling the detection of high-frequency components generated by abrupt corrections or movement oscillations. Furthermore, according to the formula described in Equation (4), the values are negative; values closer to zero indicate smoother movements, while more negative values indicate less smooth movements.

### 7.4. Statistical Analysis and Discussion

To identify biomarkers that evidenced a difference between YAs and OAs during wrist movement assessment, statistical analyses were performed on SPARC, RMSE, and the number of trajectory errors during maze execution.

A linear mixed-effects model, with group (YAs, OAs), maze level (1–3), and movement direction (F, E, R, and U) as fixed effects and subject as a random factor, was performed for SPARC analysis. The results evidenced no significant main effect of group (*p* = 0.478), indicating that movement smoothness was preserved between YAs and OAs. Mean SPARC values were (−1.576 ± 0.071) for YAs and (−1.583 ± 0.094) for OAs. Then, no significant effects at the task level or the group × level interaction were identified. However, significant directional effects were observed, suggesting that movement smoothness was influenced primarily by biomechanical and motor control demands associated with wrist movement direction.

Analysis of the RMSE demonstrated differences between YAs and OAs. Global RMSE comparison revealed differences in OAs (92.95 ± 47.81) and YAs (28.52 ± 14.37), with a statistical difference (*p* = 0.00077) and a large effect size (d = 2.06). Level analyses showed differences as task complexity increased, with significant group differences at Levels 2 (*p* = 0.0011) and 3 (*p* = 0.040), while Level 1 showed (*p* = 0.057).

On the other hand, OAs committed a larger number of errors (6.00 ± 2.83) compared to YA participants (2.17 ± 1.24), with a significant statistical difference (*p* = 0.0043) and a large effect size (d = 1.52). Level analysis demonstrated differences at Levels 1 (*p* = 0.019) and 3 (*p* = 0.013), whereas Level 2 showed a tendency toward significance (*p* = 0.067).

The results suggest that aging affects path accuracy and visuomotor control rather than movement smoothness, since OA participants maintained similar SPARC levels. However, OAs exhibited substantially larger trajectory deviations and more execution errors as task complexity increased. The results suggest partial preservation of basic motor smoothness mechanisms, alongside deterioration in sensorimotor integration and online trajectory correction processes associated with aging.

## 8. Conclusions

This work presented the results of analyzing processed kinematic signals from 10 YAs and 10 OAs. The tests involved implementing a serious video game based on a maze-solving task with three levels, used as an interactive environment to quantify wrist movement performance via a ULRR.

The results included analyses of ROM, path accuracy (RMSE), number of trajectory collisions, and movement smoothness using the SPARC. Regarding path accuracy and task execution performance, the YAs demonstrated superior performance compared to the OAs, since OAs exhibited larger trajectory deviations (92.95 ± 47.81) than YAs (28.52 ± 14.37), as well as a higher number of trajectory collisions (6.00 ± 2.83) vs. (2.17 ± 1.24). Likewise, ROM analyses revealed group differences of 2.254 for radial deviation, 3.069 for ulnar deviation, 2.660 for extension, and 0.490 for flexion movements.

In contrast, the SPARC analysis yielded mixed results, since SPARC values remained relatively similar between YAs and OAs, with differences below 0.20. This behavior may be associated with the nature of the proposed task, since continuous maze tracking and trajectory correction are likely to have induced frequent microcorrections during movement execution, generating additional high-frequency components in the velocity profile. Consequently, although SPARC did not strongly differentiate between YAs and OAs, it still provided complementary information about motor execution during complex visuomotor interaction.

Overall, the evaluated metrics enabled the identification of age-related differences in wrist motor performance, particularly regarding trajectory accuracy and task execution consistency. This work represents a pilot study focused on wrist kinematic analysis; future work will include larger sample sizes and more complex movements.

## Figures and Tables

**Table 1:** Feature significance.

Domain	Metrics	Physiological Interpretation	Clinical Relevance
Joint capacity	ROM	Usable ROM	Related to stiffness, mechanical restriction, and functional limitation
Motor performance	Path accuracy	Precision and accuracy of movement trajectory	Associated with impaired motor control and trajectory deviations in neurological and aging-related populations
Quality of movement	SPARC	Smoothness and overall coordination of movement	Longitudinally associated with the FMA for upper extremity in post-stroke populations

**Table 2:** Mean ROM values of wrist movements for OAs and YAs. ΔROM indicated the group mean difference.

Group	R (Degrees)	U (Degrees)	E (Degrees)	F (Degrees)
OAs	31.628 ± 4.950	37.066 ± 4.504	57.764 ± 15.609	61.974 ± 10.012
YAs	34.032 ± 2.160	37.339 ± 1.571	61.049 ± 12.143	61.152 ± 4.840
ΔROM	2.404	0.273	3.285	0.822

**Table 3:** Mean ± SD of RMSE values for each level in OAs and YAs. ΔRMSE indicated the group mean difference.

Group	Level 1 (Pixels)	Level 2 (Pixels)	Level 3 (Pixels)
OAs	89.674 ± 88.752	82.712 ± 34.939	106.450 ± 105.445
YAs	28.708 ± 20.086	32.465 ± 12.941	27.581 ± 14.213
ΔRMSE	60.966	50.247	78.869

**Table 4:** Mean ± SD of number of collisions for each level in OAs and YAs. ΔError indicated the group mean difference.

Group	Level 1 (Errors)	Level 2 (Errors)	Level 3 (Errors)	Mean (Errors)
OAs	5.200 ± 4.315	5.000 ± 2.944	7.800 ± 5.073	6.000 ± 1.562
YAs	1.200 ± 1.814	2.700 ± 2.263	2.600 ± 2.633	2.167 ± 0.838
ΔError	4.000	2.300	5.200	3.833

**Table 5:** Mean ± SD of wrist movement trajectories for YAs and OAs at Level 1. Δ SPARC indicated the group mean difference.

Movement	R	U	E	F
OAs	−1.552 ± 0.087	−1.592 ± 0.083	−1.582 ± 0.090	−1.569 ± 0.065
YAs	−1.573 ± 0.104	−1.6 ± 0.109	−1.586± 0.043	−1.588 ± 0.053
ΔSPARC	0.021	0.008	0.004	0.019

**Table 6:** Mean ± SD of wrist movement trajectories for YAs and OAs at Level 2. Δ SPARC indicated the group mean difference.

Movement	R	U	E	F
OAs	−1.618 ± 0.182	−1.576 ± 0.058	−1.533 ± 0.036	−1.648 ± 0.214
YAs	−1.548 ± 0.052	−1.597 ± 0.085	−1.545 ± 0.052	−1.613 ± 0.159
ΔSPARC	0.070	0.020	0.012	0.035

**Table 7:** Mean ± SD of wrist movement trajectories for YAs and OAs at Level 3. Δ SPARC indicated the group mean difference.

Movement	R	U	E	F
OAs	−1.579 ± 0.053	−1.602 ± 0.047	−1.568 ± 0.029	−1.568 ± 0.029
YAs	−1.558 ± 0.058	−1.585 ± 0.041	−1.546 ± 0.033	−1.570 ± 0.037
ΔSPARC	0.021	0.017	0.022	0.007

## Data Availability

No new data were created or analyzed in this study..
